# Novel Multidrug-Resistant *Cronobacter sakazakii* Causing Meningitis in Neonate, China, 2015

**DOI:** 10.3201/eid2411.180718

**Published:** 2018-11

**Authors:** Haiyan Zeng, Tao Lei, Wenjing He, Jumei Zhang, Bingshao Liang, Chengsi Li, Na Ling, Yu Ding, Shi Wu, Juan Wang, Qingping Wu

**Affiliations:** Guangdong Institute of Microbiology, Guangzhou, China (H. Zeng, T. Lei, W. He, J. Zhang, C. Li, N. Ling, S. Wu, Q. Wu);; Guangzhou Women and Children’s Medical Center, Guangzhou (B. Liang);; Jinan University, Guangzhou (Y. Ding);; South China Agricultural University, Guangzhou (J. Wang)

## Abstract

We report a case of meningitis in a neonate in China, which was caused by a novel multidrug-resistant *Cronobacter sakazakii* strain, sequence type 256, capsular profile K1:CA1. We identified genetic factors associated with bacterial pathogenicity and antimicrobial drug resistance in the genome and plasmids. Enhanced surveillance of this organism is warranted.

*Cronobacter sakazakii* is a foodborne pathogen associated with outbreaks of life-threatening necrotizing enterocolitis, meningitis, and sepsis in neonates and infants. Although the incidence of *C. sakazakii* infection is low, fatality rates range from 40% to 80% (*1*). Infections are usually limited to specific sequence types (STs) and complex clonal complexes (*2*–*4*). *C.* *sakazakii* ST4 is predominantly associated with meningitis in neonates; *C. sakazakii* ST12, with necrotizing enterocolitis in neonates (*2*,*3*). *C. sakazakii* is usually resistant to cephalothin and penicillin but is more sensitive to antimicrobial drugs than are other members of the family *Enterobacteriaceae*. Few reports describe drug-resistance patterns in *C. sakazakii* isolates (*4*–*6*). We report 1 multidrug-resistant (MDR) *C. sakazakii* ST256 strain that caused meningitis in a neonate in China.

On September 29, 2015, a 26-day-old boy, who was born after 38 weeks’ gestation and had abdominal distention, fever, and jaundice, was hospitalized in a children’s hospital in Guangzhou, China. He was fed breast milk; however, it could not be determined whether he had been exclusively breast-fed or whether the breast milk had been expressed by use of a pump. His cerebrospinal fluid contained numerous leukocytes, and his cerebrum contained abscesses. After a series of symptomatic treatments, including initial intravenous ceftazidime followed by meropenem, his clinical signs gradually improved. However, when discharged from the hospital after 3 weeks, his mental and physical development were remarkably impaired. 

*Cronobacter*, isolated from brain abscess fluid, was identified by using an automated VITEK 2 Compact system (bioMérieux, Marcy l’Etoile, France). An isolate, GZcsf-1, was determined to be *C. sakazakii* ST256 with serotype O1. The *Cronobacter* PubMLST (https://pubmlst.org/cronobacter/) contains 2 ST256 isolates: MOD1-Ls15 g, isolated from the alimentary canal of the green bottle fly, *Lucilia sericata*, in the United States; and 2061 (no source information), detected in France. This ST had not been reported to cause meningitis in neonates. Susceptibility to 15 antimicrobials was tested by using the broth dilution method; MICs are shown in the Technical Appendix. GZcsf-1 was resistant to 8 antimicrobials: ampicillin, cefazolin, ceftriaxone, aztreonam, gentamicin, tetracycline, chloramphenicol, and trimethoprim/sulfamethoxazole.

The Beijing Genomics Institute performed genomic and plasmid DNA sequencing by using the PacBio RS II (Pacific Biosciences, Menlo Park, CA, USA) and HiSeq (Illumina, San Diego, CA, USA) platforms. The annotations were performed as previously described (*7*). *C. sakazakii* GZcsf-1 had 1 circular chromosome, 4.43 Mb long, containing 56.87% GC and 2 plasmids (denoted pGW1, 340,723 bp, 57.2% GC; pGW2, 135,306 bp, 54.0% GC) (GenBank accession nos. CP028974–6). On the basis of the characteristics of K antigen and colanic acid biosynthesis encoding genes in GZcsf-1, we determined its capsular profile to be K1:CA1, which differs from the capsular profile K2:CA2, proposed to be strongly associated with *C. sakazakii* isolated from neonates with severe infection (*3*). The subtype I-E CRISPR-Cas system and CRISPR1 to CRISPR3 arrays were detected (*7*).

We identified mobile elements and different types of secretion systems by using VRprofile (http://202.120.12.134/STEP/STEP_VR.html) (Technical Appendix). Although virulence genes in *C. sakazakii* have not yet been clarified, T4SS, T6SS, and prophages may contribute to the pathogenicity of GZcsf-1. More importantly, full-plasmid comparison by blastn (http://blast.ncbi.nlm.nih.gov/Blast.cgi) revealed pGW2 to be closely related to IncFIB-type plasmid pESA3, which has been widely identified as a virulence plasmid in pathogenic strains of *C. sakazakii* (*8*).

We identified drug-resistance genes by using ResFinder 2.1 (https://cge.cbs.dtu.dk/services/ResFinder-2.1/). Except for 2 drug-resistance genes in the genome, 19 were integrated in pGW1, indicating that the MDR phenotype of *C. sakazakii* GZcsf-1 resulted from plasmid-mediated resistance (Technical Appendix). By performing plasmid multilocus sequence typing (https://pubmlst.org/bigsdb?db=pubmlst_plasmid_seqdef&page=sequenceQuery), we determined that pGW1 is IncHI2-ST1 and is mainly found in other countries (*9*). Full-plasmid comparison revealed that pGW1 is closely related to p505108-MDR (*5*).

The drug-resistance gene *bla_DHA-1_*, encoding an AmpC β-lactamase and conferring cephalosporin resistance, was found in pGW1 (Figure, panel A). According to a previous report, this gene is associated with ISCR1 (*10*). The difference between the 2 plasmids pGW1and p505108-MDR was in the insertion of a 7-kb fragment, associated with mercury resistance, between *sul1* and IS26 in pGW1 (Figure, panel A). This fragment was also detected in plasmid unitig_2, implying that there might be a transposition or homologous recombination. Of 19 drug-resistance genes, 9 (47.4%) were integrated in the MDR region of pGW1 (Figure, panel B). The fragment from Tn3 remnant to *mrr* gene in pGW1 was identical to that in p505108-MDR. Compared with the Tn2 remnant in p505108-MDR, only *bla_TEM-1B_* in reverse orientation was conserved in pGW1. We found 5 new drug-resistance genes in pGW1—*mph(A)*, *sul1*, *aadA2*, *dfrA12*, and *aac(**3**)-IId*—adjacent to IS26, IS5075, and ISCfr1, constituting new accessory modules. Mobilization of these accessory resistance modules into plasmid backbones may be promoted by transposition and homologous recombination. 

**Figure:**
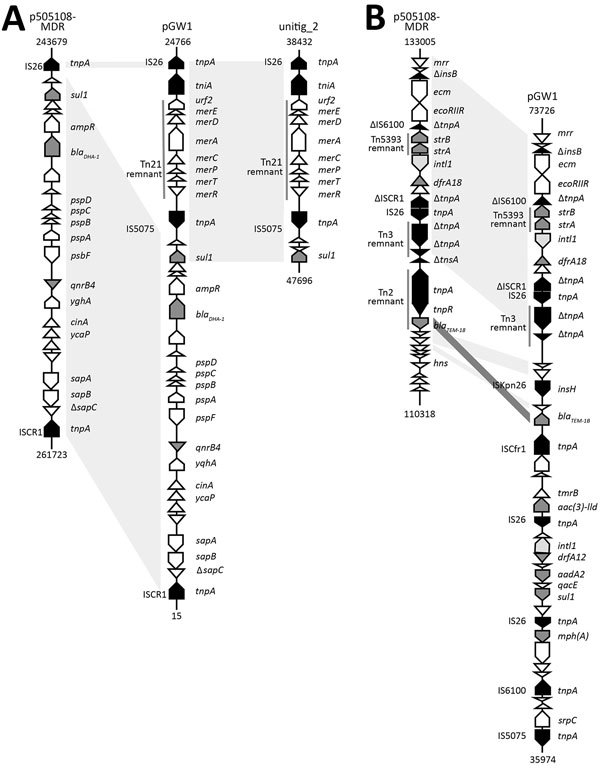
Analysis of resistance plasmid pGW1 in *Cronobacter sakazakii* sequence type 256, isolated from neonate with meningitis, China, 2015, showing genetic organization of major drug-resistance determinants in pGW1, along with its structural comparison with that of p505108-MDR. The *bla_DHA-1_* region (A) and multidrug-resistance region (B) of plasmid pGW1 are compared with those of plasmid p505108-MDR, unitig_2, or both. Black indicates genes encoding transposase, light gray indicates genes encoding integrase, dark gray indicates genes encoding drug resistance, and white indicates other genes. A few hypothetical genes with nucleotide sequences <200 bp are not shown. Shading denotes regions of homology (>95% nt identity); light gray shading indicates same orientation, and dark gray shading indicates reverse orientation. Numbers indicate nucleotide positions within the corresponding plasmids.

This study provides a new insight into *C. sakazakii* pathovars and raises concern that plasmid-mediated MDR *C. sakazakii* maybe a threat to infant health. Enhanced surveillance of antimicrobial drug–resistant *Cronobacter* is warranted.

Technical Appendix Antimicrobial drug susceptibility profiles and large mobile elements and secretion systems regions and drug resistance genes identified in *Chronobacter sakazakii* strain GZcsf-1, isolated from a neonate in China, 2015.
