# Supplementary material for: Novel Multidrug-Resistant Cronobacter sakazakii Causing Meningitis in Neonate, China, 2015
Source: Emerg Infect Dis. 2018 Nov;24(11):2121–4. doi: 10.3201/eid2411.180718 (PMC6199977; doi:10.3201/eid2411.180718)
Supplement: Technical Appendix — Antimicrobial drug susceptibility profiles and large mobile elements and secretion systems regions and drug resistance genes identified in Chronobacter sakazakii strain GZcsf-1, isolated from a neonate in China, 2015. [file 18-0718-Techapp-s1.pdf]

Article DOI: https://doi.org/10.3201/eid2411.180718

Novel Multidrug-Resistant *Cronobacter sakazakii* Causing Meningitis in Neonates, China, 2015

Technical Appendix

Technical Appendix Table 1. Antimicrobial drug susceptibility profiles

Category	Antibiotics	MIC, mg/L/antimicrobial susceptibility*
Penicillin	Ampicillin	≥32/R
Cephalosporin	Cefazolin	≥64/R
	Ceftriaxone	≥64/R
	Ceftazidime	2/S
	Cefepime	≤1/S
Carbapenem	Imipenem	≤1/S
	Ertapenem	≤0.5/S
Monobactam	Aztreonam	32/R
Aminoglycoside	Gentamicin	≥16/R
	Tobramycin	8/I
Tetracycline	Tetracycline	≥16/R
Phenicol	Chloramphenicol	≥32/R
Folate pathway Inhibitors	Trimethoprim–sulfamethoxazole	≥320/R
Nitrofuran	Nitrofurantoin	64/I
Fluoroquinolone	Ciprofloxacin	≤0.25/S

*The results were interpreted using the *Enterobacteriaceae* data from the Clinical and Laboratory Standards Institute (CLSI).

Technical Appendix Table 2. Large mobile elements and different types of secretion systems regions (>10 kb), and drug resistance genes identified in *C. sakazakii* strain GZcsf-1

Location	Type*	Nucleotide position	G+C%	Drug resistance marker	Resistance phenotype	Nucleotide position
Genome	ICE-1	530004–610181	55.0	*sul1*	Sulfonamide	595806–596645
				aadA1	Aminoglycoside	597150–597941
	T4SS	545665–566157	58.0			
	prophage-1	1033049–1074601	55.1			
	GI-1	1519846–1536984	51.1			
	ICE-2	1642519–1691107	47.5			
	prophage-2	2082823–2120897	50.1			
	GI-2	2299395–2309757	54.0			
	T6SS-1	2483935–2503145	56.6			
	T6SS-2	3176946–3204571	51.6			
	GI-3	3850021–3879063	42.7			
	T6SS-3	4360551–4404179	56.6			
pGW1				*qnrB4*	Fluoroquinolone	8018–8665
				bla_{DHA-1}	Beta-lactam	12786–13925
				sul1	Sulfonamide	15502–16341
				bla_{SFO-1}	Beta-lactam	26538–27425
				mph(A)	Macrolide	42030–42935
				sul1	Sulfonamide	44465–45304
				aadA2	Aminoglycoside	45809–46600
				dfrA12	Trimethoprim	47008–47505
				aac(3)-IId	Aminoglycoside	50075–50935
				bla_{TEM-1B}	Beta-lactam	55907–56767
				dfrA18	Trimethoprim	65766–66335
				strA	Aminoglycoside	68109–68912
				strB	Aminoglycoside	68912–69748
				aphA1	Aminoglycoside	106293–107108
				tet(D)	Tetracycline	173801–174985
				catA2	Phenicol	176597–177238
				aac(6')-IIc	Aminoglycoside	332723–333304
				ereA2	Macrolide	337620–338660
				sul1	Sulfonamide	339184–340023
pGW2	T6SS	35992–66569	55.3			

*ICE: Integrative and conjugative elements; GI: Genomic islands.
